# Nutritional requirements of meat-type and egg-type ducks: what do we know?

**DOI:** 10.1186/s40104-017-0217-x

**Published:** 2018-01-16

**Authors:** Ahmed Mohamed Fouad, Dong Ruan, Shuang Wang, Wei Chen, Weiguang Xia, Chuntian Zheng

**Affiliations:** 1 Institute of Animal Science, Guangdong Academy of Agricultural Science, Key Laboratory of Animal Nutrition and Feed Science (South China) of Ministry of Agriculture, State Key Laboratory of Livestock and Poultry Breeding, Guangdong Public Laboratory of Animal Breeding and Nutrition, Guangdong Key Laboratory of Animal Breeding and Nutrition, Guangzhou, China; 2 Department of Animal Production, Faculty of Agriculture, Cairo University, Giza, Egypt

**Keywords:** Ducks, Nutritional requirements

## Abstract

The demand for duck meat, duck eggs, and associated products is increasing each year. Classic and modern selection programs have been applied to enhance the economic traits of ducks to satisfy the requirements of consumers and enhance the incomes of producers. The nutritional requirements of unselected ducks may not be adequate, however, to fulfill the potential productivity performance of modern birds, including both meat-type and egg-type ducks. In particular, an imbalanced diet is associated with low productive performance and signs of nutritional deficiency (if insufficient nutrients are supplied), as well as with high feed costs and manure problems that reflect flock health and welfare (if excessive nutrients are supplied). Thus, the main aim of this review is to summarize the results of previous studies that estimated the nutrient requirements of meat-type and egg-type ducks in order to evaluate current knowledge and to identify further issues that need to be addressed. In addition, the results obtained in previous studies are compared in order to understand how to lower commercial feed costs, fulfill the genetic potential of selected ducks, protect the environment from pollution, and satisfy the welfare and health needs of ducks.

## Background

Globally, the production of duck meat and eggs is increasing annually. Asia is the biggest producer of duck meat and eggs [1], and China is the main producer [2]. Genetic improvement programs for meat-type ducks have successfully enhanced their productive performance. In particular, Pekin ducks are well known as the main meat-type duck and they have been genetically enhanced to obtain a higher meat yield and lower rate of carcass fat deposition (lean meat) during 6 wk to reach 3.2 kg, whereas the unimproved type requires 11 wk to reach only 1.7 kg [1, 3, 4]. In addition, many studies have aimed to improve the main egg-type breed of ducks (Longyan laying ducks), important for several reasons, viz.: medium size (1.2–1.3 kg), early maturation (110 d), high egg yield (more than 280 eggs/yr), huge populations (more than 300 million birds), adaptation to high ambient temperature, and resistance to disease [2, 5, 6]. However, the recommendations of the National Research Council 1994 (NRC1994) [7] for approving the development of a new strain of meat-type ducks, however, defined their nutritional requirements based on 40-year-old data, or on results obtained in other species. Moreover, NRC1994 [7] did not define nutritional requirements for egg-type ducks. Thus, the present review summarizes the results obtained in recent studies of both meat-type and egg-type ducks in order to determine the current nutritional requirements of ducks, as well as to provide guidance for duck producers and duck feed manufacturers.

### Protein and energy

Protein and energy are the first nutritional requirements that should be considered when formulating a diet because they represent the most expensive dietary components, but also because of their impact on the productive and reproductive performance of flocks for meat or egg production [8]. Fan et al. [9] determined the effects of the energy levels on performance and carcass quality in Pekin ducks from 2 to 6 wk of age. Their results showed that an energy level increase from 2,600 to 3,100 kcal of AME/kg had a positive effect on the productive performance, but a negative effect on the carcass quality by increasing body fat deposition. Thus, based on the growth performance, they recommended 3,000 kcal of AME/kg based on the growth performance when the diet contains 18% crude protein (CP). In addition, Xie et al. [10] suggested 2,900 kcal of AME/kg with 20.5% CP for male White Pekin ducks during the first 3 wk of age based on the growth performance and carcass quality when energy levels from 2,450 to 3,050 kcal of AME/kg were tested. Wickramasuriya et al. [11] tested increasing energy levels (2,600 to 3,300 kcal of AME/kg) and showed that native Korean ducks require 2,900 kcal of AME/kg with 18 CP% from hatching to 21 d of age in order to maximize their productive performance and carcass weight. By contrast, few studies have aimed to estimate the energy and protein needs of laying ducks. However, Thongwittaya et al. [12] examined the impacts of the energy and protein levels on the performance of growing laying ducks (Khaki Campbell × Thai Native) and recommended 2,700 kcal of AME/kg with 16.5% CP from 18 to 37 wk of age. In China, the commercially formulated diet contains 2,500 kcal of AME/kg and 17% CP to satisfy the energy and protein requirements of egg-laying ducks.

### Amino acids

Amino acids have essential roles in poultry nutrition due to their effects on performance, immunity, and meat and egg quality [8, 13]. Methionine (Met) is the first main limiting amino acid in poultry nutrition. Thus, White Pekin ducks require 0.337% Met to obtain the highest average daily body weight gain, but this amount may be adjusted 0.339% to achieve the highest breast meat proportion according to Xie et al. [14] who estimated the Met requirements of white Pekin ducks from 3 to 7 wk of age. Elkin et al. [15] reported that the highest body weight gain in White Pekin ducklings during the first 2 wk of age required a diet containing 0.338% Met, while Xie et al. [16] found that diet formulations containing 0.481% Met maximized the growth performance in Pekin ducklings during the first 3 wk of age. Zeng et al. [17] showed that White Pekin ducks, from 15 to 28 d of age, require diets containing 0.510, 0.445, and 0.404% Met to optimize their body weight gain, breast meat yield, and feather development, respectively, but for 15 to 35 d of age, these amounts were 0.468% Met to optimize the body weight gain, 0.408% Met for the breast meat yield, and 0.484% Met for feather development. In addition, Shaoxing laying ducks require 0.40% Met in their diet to maximize the egg production, egg weight, and feed conversion ratio (FCR) from 60 to 66 wk of age [18]. Fouad et al. [19] found that the egg weight, egg mass, and FCR were maximized in Longyan laying ducks from 19 to 47 wk of age by feeding a diet containing 0.41%Met.

Lysine (Lys) is the second limiting amino acid for poultry nutrition. In White Pekin ducks, Bons et al. [20] found that maximizing body weight gain required 1.06% Lys during the first 3 wk of age, for 3 to 7 wk of age, achieving the highest body weight gain, carcass yield, and breast yield required 1.02% Lys. Xie et al. [21] demonstrated that male White Pekin ducklings from 7 to 21 d of age, required 0.84, 0.90, and 0.98% Lys to achieve the highest body weight gain, feed efficiency, and breast meat proportion, respectively. Optimal body weight gain and feed efficiency were achieved with 0.71 and 1.01% Lys, respectively, in male Korean native ducklings from 1 to 21 d of age [22]. Optimizing the performance (egg production, egg weight, egg mass, and FCR) and eggshell quality in Longyan laying ducks, from 22 to 38 wk of age, requires feeding diets containing 0.80% Lys [23].

The third limiting amino acid in poultry nutrition is threonine (Thr). Zhang et al. [24], conducted two experiments to estimate the Thr requirements of White Pekin ducks from 1 to 14 d of age and diets containing 0.86, 0.92, and 0.95% Thr achieved the highest body weight gain, feed efficiency, and the maximal breast meat proportion. In addition, Xie et al. [25] recommended 0.67% Thr in the diets of White Pekin ducks from 1 to 21 d of age to maximize the body weight gain. Zhang et al. [26] suggested feeding White Pekin ducks, from 14 to 35 d of age, diets containing 0.75, 0.74, and 0.73% Thr to achieve the standardized body weight gain, FCR, and relative breast meat yield (weight), respectively. In contrast, maximizing egg production, egg mass, and feed efficiency in Longyan laying ducks requires 0.57% Thr in the diet from 17 to 45 wk of age according to previous study in this laboratory [27].

Arginine (Arg) is not classified as a limiting amino acid but it is essential for poultry nutrition because avian species are incapable of its synthesis and it is also important as a precursor substrate for generating other molecules (e.g., nitric oxide, proteins, creatine, ornithine, glutamate, polyamines, proline, glutamine, and agmatine) with various physiological functions [28]. Wang et al. [29] found that in male White Pekin ducks from hatching to 21 d of age, the optimum body weight gain, FCR, and breast meat yield expressed as breast the weight relative to live body weight required 0.95, 1.16, and 0.99% Arg, respectively. Reproductive system development in Longyan laying ducks from 17 to 31 wk of age requires feeding them diets containing 1.13% Arg, but diets containing 1.46% Arg are recommended to obtain the best egg weight with highest eggshell quality [30].

### Minerals

Calcium (Ca) in the form of calcium carbonate constitutes 96% of the eggshell, and almost 60–70% of the bone weight is Ca and P in the form of hydroxyapatite [31, 32]. Therefore, providing inadequate amounts of Ca, P, and vitamin D, as well as neglecting their optimal relative ratios in poultry diets will lead to productivity problems and economic losses. For example, Ca deficiency in laying ducks reduces the egg production rate, egg weight, eggshell thickness, eggshell breaking strength, and Ca deposition in the eggshell as a result of decreased Ca levels in the plasma and shell gland, as well as downregulating the expression of genes related to Ca transportation and eggshell biomineralization [33, 34]. In White Pekin and Mule ducks, Ca deficiency can lead to rickets, low growth rate, low feed utilization, and a high mortality rate [35, 36]. Xia et al. [37] estimated the Ca requirements of Longyan laying ducks, from 20 to 33 wk of age, and recommended formulating diets containing 3.6% Ca with 0.40% available P to maximize egg production, egg mass, feed utilization, and enhance the bone characteristics. In addition, 0.806% Ca plus 0.403% non-phytate phosphorus and 0.796% Ca plus 0.379% non-phytate phosphorus were recommended to attain the highest daily weight gain and lowest FCR, respectively, in White Pekin ducks during the first 3 wk of age [38], whereas 0.72% Ca plus 0.37% non-phytate phosphorus were suggested to optimize daily weight gain from 3 to 6 wk of age in White Pekin ducks [39].

Copper (Cu) is a growth enhancer and antimicrobial as well as being a cofactor for many enzymes, e.g., cytochrome oxidase, lysyl oxidase, tyrosinase, p-hydroxyphenyl pyruvate hydrolase, dopamine beta hydroxylase, and copper-zinc superoxide dismutase [40]. Birds fed diets deficient in Cu may suffer from hypertriglyceridemia, hypercholesterolemia, anemia, feather depigmentation, abnormal bones, and poor growth [40]. Increasing the Cu level in the diets of laying birds can improve their performance (egg production and egg weight) and egg quality (lower percentage of abnormal eggs, i.e., soft and broken eggs), as well as reducing the total cholesterol, triglycerides, and low density-lipoprotein cholesterol levels, and increasing the high density-lipoprotein cholesterol levels in the blood [41–44]. In addition, adding Cu to the diets of broiler chickens can improve the productive performance, breast meat yield, meat quality (juiciness), immunity, and the abundance of beneficial intestinal microflora (*Lactobacillus* and *Bifidobacterium*), as well as reducing the mortality rate and the abundance of harmful intestinal microflora (coliforms and *Escherichia coli*) [45, 46]. In male White Pekin ducks, Attia et al. [47] showed that improving the productive performance from hatching to 56 d of age required a diet containing 7 mg Cu/kg. Shanma laying ducks, however, require diets containing 5 mg Cu/kg from 17 to 45 wk of age to fully express their genetic potential [48]. Meat-type ducks and egg-type ducks require low amounts of Cu in their diets compared with the requirements needed to reduce the cholesterol levels in their products. Thus, increasing the Cu concentration in the diets of male White Pekin ducks to 157 mg/kg reduced the cholesterol concentration in their meat [47]. Increasing the Cu concentration in the diet beyond 120 mg/kg, however, leads to abnormal spleen, liver, and intestinal morphology in poultry [49].

Zinc (Zn) is a unique mineral that is active in many enzymes, such as copper zinc superoxide dismutase (associated with the antioxidant defense system), carbonic anhydrase (associated with supplying carbonate ions during eggshell formation), and alkaline phosphatase (associated with bone structure and calcification), as well as in the regulation of lipids, protein metabolism, and sex hormones [50–53]. Hence, diets with inadequate Zn levels can inhibit egg production, fertility, hatchability, embryo development, hatched chicken availability, feather growth, and the development and proliferation of the immune organs, as well as increasing the incidence of leg problems, oxidative damage, and the mortality rate in poultry [54–58]. In contrast, the inclusion of Zn in the diet enhances eggshell quality by increasing carbonic anhydrase secretion during the late laying period in hens [57], as well as the immune response (humoral and cellular), resistance to disease [59, 60], bone structure by increasing calcium and phosphorus accumulation (which is reflected in bone strength) [61], growth rate, carcass yield, breast meat yield by promoting protein synthesis and suppressing protein degradation [52], and meat quality (color, water holding capacity, tenderness, and taste) in broiler chickens [53], and the egg yield, egg weight, fertility, hatchability, normal number of hatched chickens, and antioxidant capacity in broiler breeders [57]. Attia et al. [62] showed that in male White Pekin ducks, maximizing the body weight gain from 1 to 56 d requires diets supplemented with 30 mg Zn/kg, whereas 120 mg Zn/kg is needed to reduce body fat deposition and improve meat quality. However, 30 mg Zn/kg in the diet is adequate for improving the performance of Longyan laying ducks during the peak laying period [63].

Manganese (Mn) is an essential trace element in poultry nutrition because of its roles in normal bone and eggshell formation, enzyme functions, and nutrient (carbohydrate and lipid) metabolism [64]. Mn activates numerous enzymes such as hydrolases, transferases, kinases, lipoprotein lipase, hormone-sensitive lipase, and mitochondrial superoxide dismutase [65, 66]. An appropriate concentration of Mn is required in poultry diets to support the endocrine system [67, 68], immune system [69], antioxidant defense system [70], bone development [71], and eggshell structure and strength [72], as well as maximizing hatchability and minimizing embryo death rate [73], while it is also beneficial for regulating body fat deposition and improving meat quality [8]. A Mn-deficient diet leads to different types of perosis, inhibits bone development, decreases egg yield, and increases the amount of eggs with poor eggshell formation [74, 75]. According to a previous study from this laboratory [76], Shanma laying ducks require diets containing 90 mg Mn/kg from 17 to 36 wk of age in order to optimize their antioxidant defense system.

Iron (Fe) is required to produce hemoglobin and to activate several enzymes, such as succinate dehydrogenase, catalase, and cytochrome c oxidase [77]. Insufficient Fe in the diet can impair body weight gains, feed consumption, FCR, and elevate mortality in broiler chickens [78], as well as reducing egg production, egg weight, and fertility rates in broiler and quail breeders [79, 80]. Dietary supplementation with Fe can increase growth rate, thymus weight, activity of antioxidant enzymes [81], and cell-mediated responses in broilers [82]. In Shanma laying ducks, Xia et al. [83] showed that 52.2, 97.2, and 127.2 mg Fe/kg are required to maintain performance, improve the yolk color, and enhance hemoglobin and hematocrit levels, respectively.

Selenium (Se) is a component of the enzyme glutathione peroxidase and it is required to produce type 1 iodothyronine deiodinase, which is needed in the thyroid gland to synthesize the active hormone triiodothyronine (T_3_) [84]. Se-deficient diets are associated with inhibited immune organ development (spleen, thymus, and bursa) [85–87], induction of oxidative injury in the liver, kidney, and pancreas (thereby affecting their vital functions) [88–90], as well as reductions in egg production, egg weight, hatchability [91], body weight gain, and feather growth [92]. By contrast, diets containing adequate levels of Se improve the egg yield, egg weight, antioxidant capacity [93, 94], meat quality (juiciness), feather growth [95], immune system defense [96], semen quality (motility and lifetime sperm number are increased, whereas the numbers of dead and abnormal sperm are decreased), fertility, and hatchability [97]. From 1 to 49 d of age, Cherry Valley hybrid ducks need 0.4 mg Se/kg to improve their growth rate [98]. In addition, Shanma laying ducks require 0.18 and 0.24 mg Se/kg of diet for egg production in the early and peak-laying phases, respectively, whereas 0.38 mg Se/kg of diet is recommended to enhance the activity of the glutathione redox system [99]. Moreover, from hatching to 3 wk of age, White Pekin ducks require 0.236 mg Se/kg of diet to protect their tissues against oxidative damage [100].

### Water-soluble vitamins

Choline is a crucial nutrient for poultry because of its role in lipid metabolism and liver functions [101]. In avian species, perosis, fatty liver syndrome, and inhibition of growth rate are induced when a diet that is deficient in choline is fed [102–104]. Thus, Lein and Jan [105] fed Tsaiya laying ducks diets supplemented with choline to investigate its effects on egg production and fatty liver syndrome. They found that adding 2,136 mg/kg of diet for only 4 wk during the peak laying period improved egg production and reduced fat accumulation in the liver. However, Ma et al. [106] found no improvement in the laying rate of Shaoxing laying ducks when fed diets supplemented with choline during their peak laying period for 20 wk, but fat deposition in the liver and the cholesterol content of eggs were reduced by adding 500 and 750 mg choline/kg of diet, respectively, although no explanation was provided for the reduction in egg cholesterol. In meat-type ducks (White Pekin ducks), Wen et al. [103, 104] found that diets containing 778, 810, and 1,182 mg choline/kg were sufficient to enhance phospholipid synthesis, improve growth rate, and prevent perosis during the first 3 wk of age, respectively, while diets containing 779 and 980 mg choline/kg were required to enhance phospholipid production and maximize the average daily weight gain from 21 to 42 d of age.

Riboflavin or vitamin B_2_ is an essential vitamin for completion of the glutathione redox cycle and generating reduced glutathione, which protect cells against oxidative damage due to its role as an endogenous antioxidant [107]. In meat-type birds, diets deficient in riboflavin can lead to growth rate retardation, leg problems, an imbalanced antioxidant defense system, low carcass yield, low meat quality, and a high mortality rate [108–112], whereas in egg-type birds, it can reduce the rates of egg production, egg weight, and hatchability [113, 114]. Tang et al. [110] demonstrated that from 1 to 21 d of age, male White Pekin ducks required diets containing 3.31 and 5.20 mg riboflavin/kg to achieve the highest daily weight gain and feed utilization rate, respectively, based on corn-corn gluten meal diets, but the amounts required were 3.27 and 3.33 mg riboflavin/kg of diet in female White Pekin ducks. In addition, male White Pekin ducks fed corn-soybean meal diets during the first 3 wk of age only required diets containing 3.01 and 2.79 mg riboflavin/kg to achieve the optimum body weight gain and FCR, respectively [111]. Similar to sex, age also affects the riboflavin requirements, where Tang et al. [112] recommended diets containing 2.43 and 2.31 mg riboflavin/kg for the optimum body weight gain and FCR, respectively, in male White Pekin ducks aged 15 to 35 d. Wang et al. [115] reported that diets containing1.32 mg riboflavin/kg satisfied the requirements for egg production, egg weight, egg mass, and FCR in Longyan laying ducks from 22 to 34 wk of age, but enhancing the Haugh unit required a diet containing 7.32 mg riboflavin.

Biotin is a coenzyme that has fundamental roles in carbohydrate, protein, and lipid metabolism [116]. Diets containing inadequate amounts of biotin can lead to fatty liver and kidney syndrome, a low hatchability rate, low growth rate, and the increased occurrence of foot pad dermatitis in birds [114–119]. Thus, injecting biotin into fertilized eggs, adding biotin to drinking water, or supplementing the diet with biotin can improve egg production, fertility, hatchability, and body weight gains, as well as decreasing the footpad burn score and hock burn score [120–123]. Zhu et al. [119] reported that diets containing 0.180 mg biotin/kg satisfy the productive performance requirements for male White Pekin ducks during the first 4 wk of age, but dietary supplementation with 0.21 mg biotin/kg is needed to minimize the number of birds suffering from foot pad dermatitis.

Folic acid is necessary for maintaining a normal growth rate and acceptable FCR in broiler chickens, as well as a greater egg weight and higher hatchability in egg-type birds [124, 125]. The egg weight and egg mass in laying hens aged 24 wk can be improved by adding 4 mg folic acid/kg of diet for 8 wk [126]. Dietary folic acid supplementation alleviates the oxidative stress induced by heat stress and improves the productive performance and carcass yield in quails [127]. Increasing the folic acid content in the diets of laying hens leads to the production of eggs that are rich in folate, which is essential for preventing many diseases in human [128]. Recently, Liu et al. [129] implicated folic acid as having a role in embryo development in broiler chickens. Moreover, Li et al. [130] found that supplementation with 150 μg folic acid in ovo enhanced the hatchability, growth performance, antibody production (IgG and IgM), and secretion of cytokines (IL-2 and IL-4) in broiler chickens. In ducks, Xia et al. [131] showed that feeding Shanma laying ducks a corn-soybean meal could meet their folic acid requirements in terms of performance from 18 to 32 wk of age, but the addition of 1.0 mg folic acid/kg to their diet was required to improve the eggshell percentage.

Pyridoxine or vitamin B_6_ deficiency inhibit feed consumption as well as promoting catabolism and retarded the growth of organs, including the comb, wattle, ovary, and veins, thereby leading to decreases in egg production, egg weight, fertility, and hatchability, as well as impaired feather growth in the offspring of mature birds [132, 133]. In broiler chickens, pyridoxine deficiency causes a high mortality rate, low body weight gain, low antibody production, and bone structure alterations, thereby leading to an increased occurrence of leg problems in flocks [134, 135]. Injecting pyridoxine into fertilized turkey eggs improves their hatchability [136]. Supplementing the diets of laying hens with 8.0 mg pyridoxine/kg improves egg production and the FCR [137]. Moreover, during the first 4 wk of age, White Pekin ducks require 2.44 mg pyridoxine to improve their growth performance [138].

Niacin or nicotinic acid is a precursor required for the biosynthesis of the coenzymes nicotinamide adenine dinucleotide and nicotinamide adenine dinucleotide phosphate, and thus it plays a fundamental role in metabolism [139]. Niacin deficiency is linked to reductions in the growth rate and feather growth, and an increased incidence of leg problems in meat-type birds [140, 141], as well as poor eggshell quality, low egg production, and decreased hatchability in egg-type birds [142]. The addition of 60 mg niacin to a corn-soybean meal diet is recommended for improving body weight gains, the meat yield, and meat quality in broiler chickens [143]. In laying hens, egg production, FCR, and eggshell quality can be improved by increasing the niacin levels [144]. In male White Pekin ducks, Xie et al. [145] found that feeding diets containing 40 mg niacin/kg from hatching until 21 d of age achieved satisfactory performance and reduced the incidence of bowed leg deformities. Wang et al. [146] recommended diets containing 90 mg niacin/kg to improve the eggshell quality in Longyan laying ducks during the early laying period.

Thiamine or vitamin B_1_ acts as a co-enzyme that participates in the oxidative decarboxylation of pyruvic acid and α-ketoglutaric acid, thereby leading to the production acetyl-coenzyme A (CoA) and succinyl-CoA, which are involved in the metabolism of carbohydrates, proteins, and lipids [116]. Feeding poultry a diet deficient in thiamine leads to reductions in appetite, growth, carcass yield, and hatchability, as well impaired carbohydrate metabolism and a high mortality rate [147–150]. A previous study in this laboratory showed that the performance of Longyan laying ducks aged 22 to 42 wk can be maximized by providing a diet containing 1.55 mg thiamine/kg [unpublished data].

### Fat-soluble vitamins

Vitamin A or retinol is essential for embryonic development, growth, reproduction, and function of the immune system. Vitamin A deficiencies lead to defects in the nervous system of embryos as well as reducing the growth rate, egg production, antibody production, and hatchability [151–154]. In broilers and broiler breeders, Yuan et al. [155], Fan et al. [156], and Chen et al. [157] showed that adequate levels of vitamin A can optimize the rates of antibody production, egg production, egg quality, and hatchability. Wei et al. [158] demonstrated that a diet containing 2,500 IU vitamin A/kg improved the productive performance in White Pekin ducks from hatching to 21 d of age. Wang et al. [159] found that a corn-soybean meal diet could satisfy the vitamin A requirements needed for performance and egg quality in Longyang laying ducks from 20 to 36 wk of age, but the addition of 400 IU vitamin A improved the antioxidant defense system.

Vitamin D and its active forms (25-hydroxycholecalciferol and 1α- hydroxycholecalciferol) affect bone development and eggshell quality because of their roles in the calcium and phosphorus cycles, but these are not their only functions in poultry. Stadelman et al. [160] reported that vitamin D deficiency reduced hatchability in breeders and the growth rate of their offspring. Aslam et al. [161] demonstrated that vitamin D deficiency retarded the growth of immune organs, and lowered the counts of macrophages and decreased their capacity for phagocytosis. Supplementation with vitamin D_3_ or it active forms improves the productive performance, breast meat yield, meat quality (by enhancing the skin and meat color, juiciness and tenderness) [162–164], immune system defense [165, 166], egg production, egg weight, and hatchability [167]. Wang et al. [168] showed that diets containing 1,000 IU vitamin D_3_/kg are sufficient to meet the needs of White Pekin ducks during the first 2 wk of age. In egg-laying ducks, Xie and Wang [169] showed that diets containing 550 IU vitamin D/kg are sufficient for Jinding ducks from hatching until 4 wk of age based on improvements in the relative weights of the immune organs and the suppression of lipid peroxidation. In an experiment from 16 to 18 wk of age, the rates of egg production, egg weight, FCR, and egg quality were not changed by dietary supplementation of vitamin D_3_ in Longyan laying ducks, but adequate bone mineralization required a diet containing 800 IU/kg [170]. Chen [171] found that optimized egg production and enhancing eggshell quality required diets containing vitamin D_3_ at 1,069 and 989 IU/kg, respectively, in Tsaiya egg-ducks from 30 to 50 wk of age.

Vitamin E protects cell membranes against damage by free radicals due to its antioxidant properties. Vitamin E is linked to the economic traits of poultry, including fertility, hatchability, immunity, and meat and egg quality. Vitamin E deficiency impairs egg production, fertility, and hatchability [172], suppresses development of immune organs [173], and leads to the occurrence of muscular dystrophy in breast muscles [174]. Thus, dietary supplementation with vitamin E enhances egg production, egg quality, fertility, semen quality (improved viability, motility, and concentration, and a reduced percentage of dead and abnormal sperm) [175–177], humoral and cell-mediated immune responses [178], meat quality by reducing drip losses and enhancing tenderness, and intramuscular fat [179]. Despite the importance of vitamin E for poultry nutrition, only one study has investigated the influence of dietary vitamin E supplementation on the performance of Tsaiya laying ducks [180]. In this study, Chen and Hsu [180] recommended dietary supplementation with 400 mg α-tocopherol/kg to enhance egg production, egg mass, and FCR in Tsaiya laying ducks from 30 to 36 wk of age.

## Conclusions

**Fig. 1:**
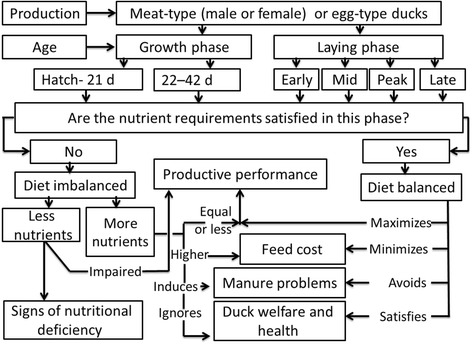
Impacts of nutritional requirements on productive performance, feed cost, manure problems, and duck welfare and health

Clearly, nutritional deficiencies can directly affect the productive performance of poultry, including ducks. The symptoms of nutritional deficiencies sometimes interact with each other, which can lead to difficulties interpreting these symptoms. However, it is not always advisable to simply increase the nutrient levels in the diet because increasing these levels may waste money and lead to losses via manure. Thus, simply increasing the amounts of proteins, amino acids, Ca, P, and other nutrients to meet the requirements of animals is not recommended because there is a lack of information and suitable studies regarding the optimal levels of these nutrients. Thus, uninformed dietary supplementation with nutrients may affect the welfare and health of animals, and increase environmental pollution. Therefore, based on the previous studies discussed above as well as the nutrient levels used in commercial feed for White Pekin ducks and Longyan laying ducks (Table 1) (the most studied strains of ducks) and considering age and gender (only in meat-type ducks), as shown in Fig. 1, it is possible to revise and modify the commercial nutritional requirements for ducks. Thus, the current situation can be evaluated and further experiments may be designed to identify the optimal requirements for duck production. This information may facilitate improvements to industrial duck production by considering duck welfare and health, as well protecting the environment from contamination with excess nutrients.

**Table 1:** Nutritional requirements of meat-type (White Pekin ducks) and egg-type ducks (Longyan ducks) in commercial production

Nutrient	1–21 d	22–42 d	Laying period
Metabolizable energy, kcal/kg	2,900	3,000	2,500
Protein, %	20	18	17
Methionine, %	0.48–0.50	0.47–0.50	0.40
Lysine, %	1.1	1.0	0.80
Threonine, %	0.70–0.80	0.70–0.80	0.60
Tryptophan, %	0.23	0.23	0.21
Calcium, %	0.83	0.89	3.60
Available phosphorus, %	0.40	0.40	0.35
Manganese, mg/kg	80–100	80–100	90
Zinc, mg/kg	60	60	90
Iron, mg/kg	60	60	50
Copper, mg/kg	10	10	10
Iodine, mg/kg	0.2	0.2	0.50
Selenium, mg/kg	0.3	0.3	0.40
Vitamin A, IU	10,000	8,000	12,000
Vitamin D_3_, IU	3,000	3,000	2,000
Vitamin E, mg/kg	20.0	20.0	38.0
Vitamin K, mg/kg	2.0	2.0	1.0
Thiamine, Vitamin B_1_, mg/kg	2.0	2.0	3.0
Riboflavin, Vitamin B_2_, mg/kg	10.0	8.0	9.6
Pyridoxine, Vitamin B_6_, mg/kg	4.0	4.0	6.0
Cyanocobalamin, Vitamin B_12_, mg/kg	0.02	0.02	0.03
Choline, mg/kg	1,000	750	500
Pantothenic acid, mg/kg	20.0	10.0	28.5
Folic acid, mg/kg	1.0	1.0	0.6
Biotin, mg/kg	0.2	0.2	0.15
Niacin, mg/kg	50.0	50.0	25.0

## Abbreviations

AME: Apparent metabolizable energy
Ca: Calcium
CP: Crude protein
Cu: Copper
Fe: Iron; Se: Selenium
Mn: Manganese
NRC: National research council
P: Phosphorus
Zn: Zinc
